# Inter-Observer and Intra-Observer Reliability Assessment of the Established Classification Systems for Periprosthetic Shoulder Fractures

**DOI:** 10.3390/jcm12093168

**Published:** 2023-04-28

**Authors:** Mats Wiethölter, Doruk Akgün, Fabian Plachel, Marvin Minkus, Daniel Karczewski, Karl Braun, Kathi Thiele, Luis Becker, Ulrich Stöckle, Philipp Moroder

**Affiliations:** 1 Department of Trauma, Hand and Reconstructive Surgery, University Hospital Münster, 48149 Münster, Germany; 2 Center for Musculoskeletal Surgery, Charité—University Medicine Berlin, 13353 Berlin, Germany; 3 Department of Trauma Surgery, Klinikum Rechts der Isar, Technical University of Munich, 81675 Munich, Germany; 4 Schulthess Klinik, 8008 Zurich, Switzerland

**Keywords:** periprosthetic shoulder fractures, shoulder arthroplasty, classification, reliability, treatment recommendation

## Abstract

This study evaluated the reliability and comprehensiveness of the Unified classification system (UCPF), Wright & Cofield, Worland and Kirchhoff classifications and related treatment recommendations for periprosthetic shoulder fractures (PPSFx). Two shoulder arthroplasty specialists (experts) and two orthopaedic residents (non-experts) assessed 20 humeral-sided and five scapula-sided cases of PPSFx. We used the unweighted Cohen’s Kappa (κ) for measuring the intra-observer reliability and Krippendorff’s alpha (α) for measuring the inter-observer reliability. The inter-rater reliabilities for the Wright & Cofield and Worland classifications were substantial for all groups. The expert and non-expert groups for UCPF also showed substantial inter-rater agreement. The all-rater group for the UCPF and the expert and non-expert group for the Kirchhoff classification revealed moderate inter-rater reliability. For the Kirchhoff classification, only fair inter-rater reliability was found for the non-expert group. Almost perfect intra-rater reliability was measured for all groups of the Wright & Cofield classification and the all-rater and expert groups of the UCPF. All groups of the Kirchhoff and Worland classifications and the group of non-experts for the UCPF had substantial intra-rater reliabilities. Regarding treatment recommendations, substantial inter-rater and moderate intra-rater reliabilities were found. Simple classification systems for PPSFx (Wright & Cofield, Worland) show the highest inter- and intra-observer reliability but lack comprehensiveness as they fail to describe scapula-sided fractures. The complex Kirchhoff classification shows limited reliability. The UCPF seems to offer an acceptable combination of comprehensiveness and reliability.

## 1. Introduction

Periprosthetic shoulder fractures (PPSFx) have become a more frequent clinical challenge due to rising numbers of implantations in an aging population [1,2,3]. The incidence of PPSFx is reported between 0.6% and 19.4% [4,5,6,7,8,9,10,11,12,13,14,15]. The appropriate therapy for PPSFx can be a challenging task for the orthopaedic surgeon [16]. Therefore, a highly reliable classification system and a clear treatment recommendation are necessary.

The literature regarding intra-rater and inter-rater reliability for PPSFx shoulder classifications is still scarce. Previous studies evaluated the reliability of classification systems for humeral-sided PPSFx [17,18,19,20]. Kuhn et al. and Auran demanded a more reliable and clinically relevant classification system [17,18].

Therefore, this study is the first that evaluated the intra- and inter-rater reliability of the established PPSFx classification systems, including the Wright & Cofield classification, Worland classification, UCPF and Kirchhoff classification, and verified whether surgeons generate consistent treatment recommendations for humeral-sided and scapula-sided PPSFx.

## 2. Materials and Methods

Twenty-seven cases of PPSFx were found in our database treated between 2011 and 2020 in our hospital. Two cases were excluded because they just had a CT scan with no X-rays found in the data bank. In total, twenty-five cases, twenty humeral-sided and five scapula-sided, were included in the study. The inclusion criteria for the study were a conservative or operative treatment for PPSFx at our hospital and the availability of x-ray images showing the extension of the fracture and the inserted arthroplasty in at least two planes (true-a.p. and y-view). The exclusion criterion was the incompleteness of the data. 

The study was approved by the Institutional Review Board of the Charité—University Medicine Berlin (ethics proposal number EA4/096/20). 

All recorded imaging data were used to create clinical case vignettes for each patient. Each case vignette was evaluated at the end of the recruitment period by four independent raters, two experienced shoulder surgeons (experts: P.M.; K.T.) and two orthopedic residents (non-experts: D.A.; M.M.). All case vignettes were anonymized and provided to the evaluators in random sequential order. The vignettes were then subsequently re-evaluated by all four raters four weeks later, again in random order, to reduce the potential recall bias. The raters were asked to classify each case according to Wright & Cofield Classification, Worland Classification, Kirchhoff Classification and UCPF and to give a treatment recommendation. The rater’s treatment recommendations were divided into four groups: (1) Conservative therapy, (2) Open Reduction and Internal Fixation (ORIF), (3) Exchange of the prosthesis and (4) a combination of ORIF and Exchange.

### 2.1. Classification of Wright & Cofield

**Figure 1:**
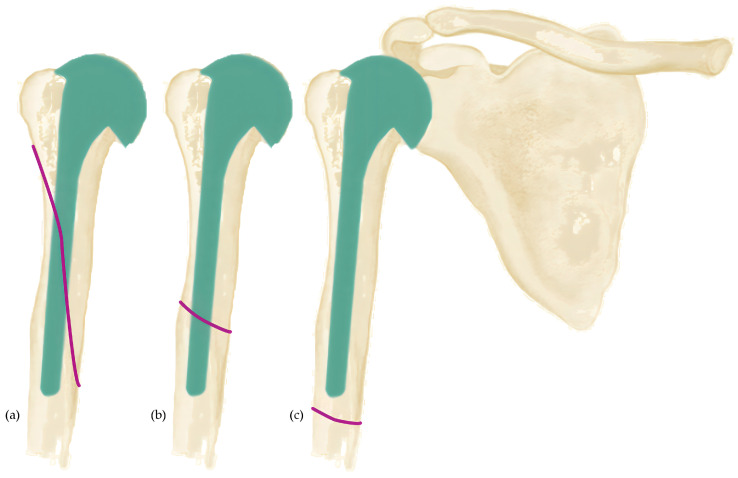
Classification of Wright & Cofield [12]. Purple lines show fracture extensions. (**a**) Type-A-fracture: around the tip of the prosthesis stem with extension proximally by more than one-third of the length of the prosthesis stem. (**b**) Type-B-fracture: localized at the tip of the prosthesis with distal extension. (**c**) Type-C-fractures: localized distal to the tip of the prosthesis stem.

In 1995, Wright and Cofield published a simple three-step classification that categorizes humeral periprosthetic fractures by their relation to the tip of the humeral stem [12]. Type-A-fractures are defined as fractures around the tip of the prosthesis with an extension of more than one-third of the prosthesis length. Type-B-fractures are also located in the area of the tip of the prosthesis but have a smaller extension proximally or can also run out distally. Fractures distal to the prosthesis stem are classified as Type-C-fractures. The classification, therefore, only refers to the localization and height of the fracture in relation to the length of the stem (Figure 1). 

### 2.2. Classification of Worland

**Figure 2:**
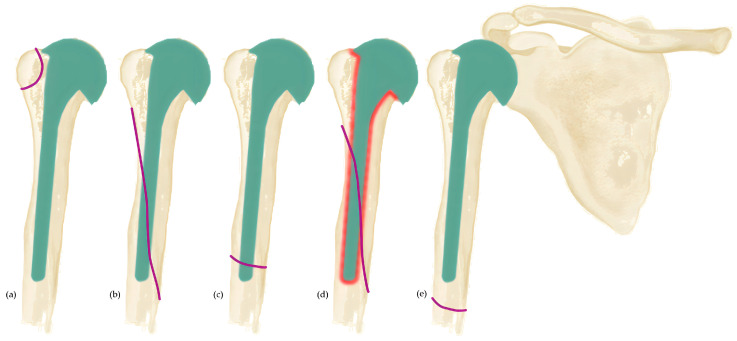
Classifications of Worland et al. [11]. Purple lines show fracture extensions. (**a**) Type-A-fracture: includes fractures of the tubercula; (**b**–**d**) Type-B-fractures: fractures in the shaft region. (**b**) Type-B1-fracture: a spiral shaft fracture with a stable shaft. (**c**) Type-B2-fracture: a transverse fracture with a stable shaft. (**d**) Type-B3-fracture: in the shaft area with a loosened prosthesis shaft. (**e**) Type-C-fracture: located sufficiently far distal to the prosthesis shaft.

The classification by Worland et al. included not only the localization but also the stability of the inserted prosthesis [11]. There is a similarity to the Vancouver classification for periprosthetic proximal femoral fractures [21]. Worland et al. divided the fractures into type A, type B, having the subgroups B1 to B3 and type C. Type-A-Fractures include fractures in the area of the tuberosity. Fractures around the shaft of the humerus are classified as type B fractures. Type B1 describes a spiral shaft fracture with a fixed prosthesis. A transversal fracture with a fixed prosthesis is defined as Type B2. Type-B3-Fractures are located in the area of the stem with a loose implant. Worland et al. recommend conservative therapy or ORIF for Type A, Type B1 and Type C fractures [11]. For Type B2 and Type B3 fractures, a change to a long stem prosthesis is indicated [11] (Figure 2).

### 2.3. Unified Classification System for Periprosthetic Fractures (UCPF)

According to the AO fracture classification, Duncan and Haddad developed the Unified classification system for periprosthetic fractures (UCPF) [22,23]. UCPF is not limited to a single joint and is a method for classifying periprosthetic fractures of the shoulder, elbow, hand, hip, knee and ankle joints. According to UCPF, every joint has a Roman numeral, and the shoulder joint is defined as No. I. The following Arabic numeral represents the fractured bone adjacent to the arthroplasty, in the case of the shoulder joint, the humerus (I.1) or glenoid/scapula (I.14). Periprosthetic fractures are divided into seven types from A to F with corresponding subgroups. A-fractures are apopyseal or extraarticular/periarticular, including a fracture of the greater (I.1.A1) or lesser tuberosity (I.1.A2) regarding the humerus. In the case of the scapula, it is an avulsion of the coracoid process (I.14.A1) or an avulsion of the acromion (I.14.A2). B-fractures are in the bed or around the implant and divided into three subgroups: B1 means a stable implant and good bone quality, B2 a loose implant and good bone quality and B3 a loose prosthesis and poor bone quality/bone defects respecting the humeral (I.1.B1–3) or glenoid implants (I.14.B1–3). C-fractures are clear of or distant to the implant. Regarding the humerus, these include a fracture of the shaft or distal (I.1.C) and a body fracture of the scapula (I.14.C). D-fractures divide the bone between two implants or are interprosthetic. Regarding the shoulder, these include, for example, between a shoulder and elbow arthroplasty (I.1.D). E-fractures according to the UCPF offer fractures of each of the two bones supporting the arthroplasty, and therefore include fractures of the humerus and scapula at the same time (I.1.E). Fractures facing and articulating with a Hemi-arthroplasty are F-fractures. With reference to the shoulder are fractures of the glenoid in contact with the inserted humeral hemiarthroplasty (I.14.E).

In 2020, Stolberg-Stolberg et al. published a treatment recommendation according to the UCPF subclassifications [24].

### 2.4. Classification of Kirchhoff

**Figure 3:**
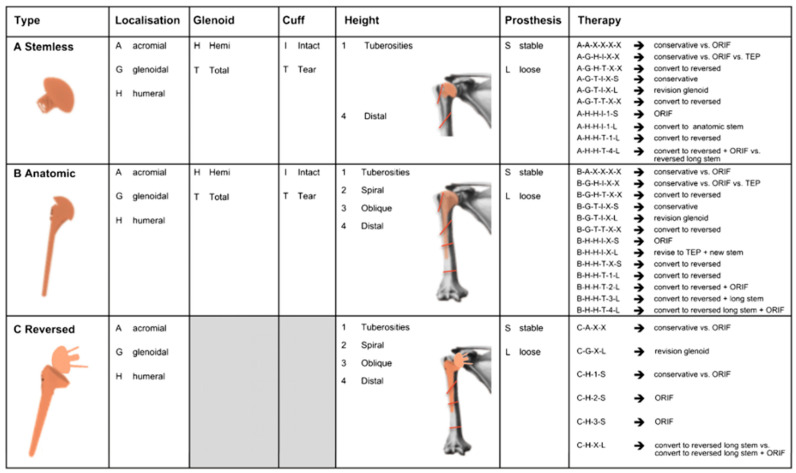
Kirchhoff classification for periprosthetic shoulder fractures [19].

The classification of Kirchhoff et al. is based on CT Imaging [25]. According to their classification, Kirchhoff et al. published a treatment recommendation in 2018 [19]. Six items specify the PPSFx: (1) type of prosthesis (stemless, anatomic, reversed); (2) fracture location as humeral, glenoidal or acromial; (3) hemi or total arthroplasty; (4) intact or torn rotator cuff; (5) relation of the fracture to the specific stem type; and (6) implant stability (loose or stable). This results in a code with six digits, from which a recommendation of treatment can be determined [25] (Figure 3). 

### 2.5. Statistical Analysis

The sample size explains that PPSFx is a rare pathology. We retrospectively reviewed all periprosthetic fractures of the shoulder joint treated in our hospital from 2011 to 2020 and included the cases if the inclusion criteria were fulfilled. 

Statistical analysis was performed using SPSS 27 (SPSS Inc., Chicago, IL, USA).

All data were analyzed using Krippendorff’s alpha (α) statistic to measure the level of inter-observer agreement for two or more observers and the unweighted Cohen’s Kappa (κ) to measure the intra-observer agreement applying the classification systems and specified treatment recommendations. The Landis and Koch criteria were used for interpretation [20]. Values of 0.00 to 0.20 indicate slight agreement; 0.21 to 0.40, fair agreement; 0.41 to 0.60, moderate agreement; 0.61 to 0.80, substantial agreement. Values of more than 0.80 represent almost perfect agreement [26].

## 3. Results

### 3.1. Comprehensiveness 

The Wright & Cofield classification and Worland classification represent only periprosthetic humerus fractures and therefore only 20 (80%) of the 25 fractures evaluated in this study. All 25 fractures tested could be evaluated by UCPF and Kirchhoff classification.

### 3.2. Inter-Rater Reliability

The inter-rater reliability of all raters for the Wright & Cofield classification was substantial (α: 0.78). No difference between the expert group (α: 0.79) and non-expert group (α: 0.79) was shown. Relating to the Worland classification, the inter-rater reliability for all raters was substantial (α: 0.65). A higher inter-rater reliability in the non-expert group (α: 0.74) compared to the expert group (α: 0.69) was shown. We found moderate inter-rater reliability for the UCPF for all raters (α: 0.57). No large difference between the substantial agreements for the group of experts (α: 0.64) and non-experts (α: 0.62) could be demonstrated. Applying the Kirchhoff classification for all raters, a moderate inter-rater agreement was found (α: 0.45). In the expert group, a slightly higher agreement (α: 0.54) was observed compared to the non-expert group (α: 0.40). The data for all groups for inter-rater reliability are given in Table 1.

### 3.3. Intra-Rater Reliability

According to the Wright & Cofield classification, an almost perfect intra-rater reliability for all raters was found (κ: 0.86). There was no substantial difference between the group of experts (κ: 0.86) and non-experts (κ: 0.85). For the Worland classification, substantial intra-rater reliability of all raters (κ: 0.79) was determined. There was a slight difference between the group of experts (κ: 0.77) and non-experts (κ: 0.80). The UCPF showed an almost perfect intra-rater reliability (κ: 0.82) for all raters. The expert group showed a higher, almost perfect intra-rater reliability (κ: 0.86) compared to the substantial agreement of the non-expert group (κ: 0.77). The intra-rater reliability using the Kirchhoff classification for all raters was substantial (κ: 0.66). The non-expert group showed a slightly higher, substantial intra-rater agreement (κ: 0.70) compared to the expert group (κ: 0.62). The data for intra-rater reliability is given in Table 2.

### 3.4. Reliabilities of Treatment Recommendations 

Applied to the treatment recommendations, moderate intra-rater reliability for all raters (κ: 0.58) was found. A higher, substantial intra-rater agreement was found for the group of experts (κ: 0.66) compared to the moderate agreement for the group of non-experts (κ: 0.50). A moderate inter-rater reliability of the specified recommendations for all raters was found (α: 0.58). The inter-rater agreement was higher for the expert group (α: 0.59) than the non-expert group (α: 0.47).

In Table 3, the intra-rater, and in Table 4, the inter-rater reliabilities for the treatment recommendation are presented.

## 4. Discussion

Maurice E. Müller said, “A classification is useful only if it considers the severity of the bone lesion and serves as a basis for treatment and for evaluation of the results [27].” A comprehensive classification with high validity, reliability and objectivity is elementary for a focused therapy. This study evaluated the intra-rater and inter-rater reliability of the established classification systems of PPSFx and analyzed if there were consistent therapy recommendations among surgeons. 

Regarding the Wright & Cofield and Worland classifications, we found substantial inter-rater and almost perfect or substantial intra-rater agreements for the tested groups. We found using UCPF moderate or substantial inter-rater and substantial and almost perfect intra-rater reliabilities. Applying the Kirchhoff classification, fair or moderate inter-rater and moderate or substantial intra-rater reliabilities were measured. This study assessed substantial inter-rater and moderate intra-rater agreements for predefined treatment recommendations. 

In 2013, Andersen et al. published an evaluation of the Wright & Cofield classification by using 36 post-operative cases of PPSFx of the humerus. Three upper extremity-trained surgeons graded on two different occasions. They reported fair inter-observer (κ: 0.37) and substantial intra-observer reliabilities (κ: 0.69) [20]. In the study of Kuhn et al. from 2022, four observers rated 34 cases of periprosthetic humeral fractures for the Wright & Cofield, Campbell, Worland and Groh classifications, and treatment recommendations encrypted and randomized on two separate occasions. The statistical analysis was slightly different but comparable to our study [17]. Kuhn et al. showed substantial intra-observer (κ: 0.703) and moderate inter-observer agreements (Fleiss’ κ: 0.583) for the Wright & Cofield classification [17]. In 2022, Auran et al. published an analysis of the intra- and inter-reliabilities of the Wright & Cofield classification, the UCPF and recommended treatment for seventy-six cases rated by three upper extremity-trained surgeons [18]. They determined a moderate intra-rater agreement (κ: 0.4.4) and a slight inter-rater agreement (κ: 0.04) for the Wright & Cofield classification [18].

Our study showed higher, almost perfect intra-observer (κ: 0.86) and substantial inter-observer agreements (α: 0.78) using the three-step classification of Wright & Cofield. 

Applying the Worland classification, Kuhn et al. evaluated fair intra-observer agreement (κ: 0.637) and moderate inter-observer reliability (Fleiss’ κ: 0.496) [17]. Higher, substantial intra-observer reliability (κ: 0.79) and higher, substantial inter-observer reliability (α: 0.65) were found in our study. The Worland classification was the gold standard to classify PPSFx before using the UCPF at our hospital; therefore, high reliabilities could be explained in addition to the quality of the X-rays. 

Auran et al. showed in their study applying the UCPF for PPSFx of the humerus substantial intra-rater reliability (κ: 0.51) and fair inter-rater reliability (κ: 0.29) [18]. In our study, we found higher, almost perfect intra-rater agreement (κ: 0.82) for grading PPSFx of the humerus and scapular. This difference may be attributed to the fact that, in our hospital, the UCPF is the standard to classify PPSFx, and therefore there may have been more experience in its use.

Kirchhoff et al. showed in the validation of their classification for PPSFx an almost perfect inter-rater reliability of two rating trauma surgeons (κ: 0.94) [19]. Our study showed a far lower, moderate inter-rater agreement for all four observers (α: 0.45), respectively for the group of experts (α: 0.54) and non-experts (α: 0.40). Kirchhoff et al. evaluated CT images, which allowed a highly probable, better assessment of the fracture and especially of the rotator cuff status than in this study, which only used X-rays for evaluation. In addition, the Kirchhoff group developed this complex classification system, so it can be assumed that they have more experience in its application [19].

Kuhn et al. showed a moderate inter-observer agreement (κ: 0.490) for preferred management strategy of PPSFx [17]. For the treatment recommendation, Auran et al. found moderate intra-rater (κ: 0.57) and moderate inter-rater observer agreement (κ: 0.41) [18]. Our study underlines these results by finding moderate intra-rater (κ: 0.58) and moderate inter-rater agreement (α: 0.49). Therefore, the decision for therapy only justified by X-rays does not seem permissible. Based on the UCPF, a treatment algorithm was described by Stolberg-Stolberg et al. [24]. The authors of the Kirchhoff classification developed an algorithm for their classification [19,25]. There are also other non-classification-based treatment algorithms for periprosthetic humerus fractures [3,5,28]. No treatment algorithm was proven superior in terms of clinical outcome score. Several retrospective studies on the outcome after therapy of periprosthetic humerus fractures with small numbers of cases have been published [10,11,12,16,20,29,30,31,32,33,34,35]. There is an urgent need for studies with larger numbers of cases for the validation of the established classification systems and classification-based outcomes after treatment to find the best possible and comparable treatment for our patients. 

As expected, the more complex classifications have lower intra-rater and inter-rater reliabilities. Generally, there are no major differences between the expert and the non-expert groups. Quick learnability without distinct previous knowledge of shoulder surgery seems to be given for all tested classification systems. The higher intra-rater reliability using the UCPF, as well as higher inter-rater reliability using the Kirchoff classification, suggests a slightly more reproducible application among the experienced shoulder surgeons for the more complex classifications. The moderate intra-rater and inter-rater reliabilities for the pre-specified recommended treatment in all publications show the need for more precise treatment recommendations for PPSFx, adjusted by more information such as cross-sectional imaging and general health status of the patient [17,18].

A limitation of this study is the small sample size, which is caused by the still low incidence of the pathology. Not every possible subtype of the tested classification systems was presented. Not every tested classification system included every presented fracture. The classification systems of Worland and Wright & Cofield only address humeral-side fractures and are therefore not comprehensive. The Kirchhoff classification is established as a CT classification; we used radiographs in our study. Audigé, Bhandari and Kellam defined a standard for the evaluation of reliability [36]. Our study fulfils many of the required characteristics, such as a clear definition of the classification systems, explicit inclusion and exclusion criteria and adapted statistical methods. The small number of observers and the lack of intraoperative findings are considered limitations. Some evaluating surgeons treated the patients and may have remembered the cases from the X-rays. With the division of the treatment recommendations into four groups, we have made a preselection, and further subdivisions and resulting lower reliabilities are possible. 

## 5. Conclusions

Our study shows that the more complex classification systems for PPSFx (UCPF, Kirchhoff) offer lower intra-observer and inter-observer reliabilities but are comprehensive. Simple classification systems (Wright & Cofield, Worland) show the highest inter- and intra-observer reliability; however, they lack comprehensiveness as they fail to describe scapula-sided fractures. The UCPF seems to offer an acceptable combination of comprehensiveness and reliability. 

Even pre-specified treatment recommendations reveal just moderate intra- and inter-observer agreements. Therefore, decision-making just including X-rays seems not acceptable. More information is needed. The number of studies on classification-based outcome measurement is still low. Hence, there is an urgent need for this kind of study. 

## Figures and Tables

**Table 1:** Inter-rater reliability.

Classification/Group	Wright & Cofield (α)	Worland (α)	UCPF (α)	Kirchoff (α)
Experts	0.79 (0.70–0.86)	0.69 (0.61–0.77)	0.64 (0.54–0.73)	0.54 (0.44–0.63)
Non-experts	0.79 (0.70–0.86)	0.74 (0.64–0.82)	0.62 (0.52–0.70)	0.40 (0.30–0.48)
All Raters	0.78 (0.74–0.82)	0.65 (0.62–0.70)	0.57 (0.52–0.61)	0.45 (0.41–0.50)

**Table 2:** Intra-rater reliability.

Classification/Group	Wright & Cofield (κ)	Worland (κ)	UCPF (κ)	Kirchhoff (κ)
Expert 1	0.95 (0.84–1.05)	0.80 (0.62–0.98)	0.95 (0.84–1.05)	0.77 (0.59–0.94)
Expert 2	0.78 (0.58–0.98)	0.75 (0.43–0.85)	0.78 (0.59–0.98)	0.48 (0.27–0.69)
Experts	0.86 (0.71–1.01)	0.77 (0.59–0.96)	0.86 (0.72–1.01)	0.62 (0.43–0.82)
Non-expert 1	0.87 (071–1.04)	0.77 (0.59–0.96)	0.90 (0.76–1.03)	0.71 (0.52–0.90)
Non-expert 2	0.83 (0.69–1.02)	0.83 (0.65–1.01)	0.64 (0.43–0.85)	0.70 (0.50–0.90)
Non-experts	0.85 (0.68–1.03)	0.80 (0.62–0.98)	0.77 (0.60–0.94)	0.70 (0.51–0.89)
All Raters	0.86 (0.69–1.02)	0.79 (0.60–0.97)	0.82 (0.66–0.98)	0.66 (0.47–0.86)

**Table 3:** Intra-rater reliability for treatment recommendations.

Value/Group	Cohens Kappa (κ)
Expert 1	0.70 (0.59–0.81)
Expert 2	0.62 (0.49–0.75)
Experts	0.66 (0.54–0.78)
Non-expert 1	0.55 (0.44–0.66)
Non-expert 2	0.45 (0.49–0.75)
Non-experts	0.50 (0.47–0.71)
All raters	0.58 (0.51–0.75)

**Table 4:** Inter-rater reliability for treatment recommendations.

Value/Group	k-Alpha (α)
Experts	0.59 (0.47–0.69)
Non-experts	0.47 (0.35–0.58)
All Raters	0.49 (0.43–0.54)

## Data Availability

The data presented in this study are available on request from the corresponding author.
